# TMEM251, a new player in lysosomal enzyme trafficking

**DOI:** 10.1093/lifemeta/loac039

**Published:** 2022-12-17

**Authors:** Liming Wang, Han-Ming Shen

**Affiliations:** School of Biomedical Sciences, Hunan University, Changsha, Hunan 410082, China; Faculty of Health Sciences, Ministry of Education Frontiers Science Center for Precision Oncology, University of Macau, Macau 999078, China

In recent studies published in *Science* and *Nature Communications*, three independent groups identified TMEM251, one transmembrane protein in Golgi, as an indispensable factor for lysosomal enzyme trafficking. Loss or mutation of TMEM251 results in hypersecretion of lysosomal enzymes due to lack of mannose-6-phosphate (M6P) modification, leading to lysosomal dysfunction and eventually lysosome storage disorders (LSDs).

The lysosome is a membrane-bound organelle discovered by Christian de Duve in the 1950s. The critical function of lysosome is to degrade and recycle intracellular materials via autophagy and extracellular materials via endocytosis as well as phagocytosis [1]. In particular, the lysosomes work as the terminal degradation stations for all types of autophagy (macroautophagy, microautophagy, and chaperone-mediated autophagy) [2]. As the key digestive organelle, lysosomes are characterized by an acidic lumen containing over 60 luminal enzymes (hydrolases) which are responsible for breaking down substrates including proteins, lipids, glycogen, etc. It is well known that most lysosomal enzymes utilize the (M6P) residues as a sorting tag for trafficking to the endosomal/lysosomal system.

The M6P-mediated lysosomal enzyme sorting has been extensively studied [3]. The lysosomal proenzymes are firstly synthesized in the endoplasmic reticulum, and then transported into the Golgi apparatus. At the cis-Golgi, the N-acetylglucosaminyl-1-phosphotransferase (GNPT) transfers N-acetylglucosamine (GlcNAc)-1-phosphate from the substrate UDP-GlcNAc onto the 6-hydroxyl of a mannose residue of lysosomal proenzymes. GNPT is a heterohexameric complex consisting of three subunits (α2β2γ2), which is cleaved and activated by site-1-protease (S1P). Next, the uncovering enzymes at the trans-Golgi cleaves the GlcNAc molecule and leaves behind the M6P monoester. Then, these lysosomal proenzymes are sorted and recognized by M6P receptors (MPRs) at the trans-Golgi. MPRs traffic between trans-Golgi and endosomes, thus delivering the enzymes to the endosomal/lysosomal system. Upon arrival in the lysosomes, the lysosomal proenzymes undergo sequential cleavage to form the mature enzymes. However, it remains elusive whether additional critical regulator(s) of M6P modification exists.

Recently, three independent groups utilized genome-scale CRISPR/Cas9 knockout screen and demonstrated that TMEM251, one transmembrane protein in Golgi, is an indispensable regulatory factor of M6P modification and plays crucial roles for lysosomal enzymes trafficking [4–6]. TMEM251 is a less characterized protein with two isoforms (a long isoform and a short isoform). Previous studies reported that TMEM251 is possibly involved in cholesterol homeostasis [7, 8] and autophagy regulation [9]. Notably, mutations in TMEM251 cause skeletal dysplasia syndromes, similar to LSDs [10]. To identify the essential regulators of lysosomal homeostasis in virus such as SARS-CoV-2 infection, Richards *et al*. performed the CRISPR/Cas9 screening in human glioblastoma cells which are susceptible to cell death following virus productive infection [4]. They used cell death as a readout and found that TMEM251 is an essential factor for diverse viruses’ infection. Knockout of TMEM251 causes severe defect of lysosomal cathepsin proteases, reduces virus production, and protects against cell death. Next, they used quantitative proteomic approaches and found that in TMEM251 knockout cells, most of lysosomal proenzymes lack M6P modification and are mis-secreted into the medium. Mechanistically, TMEM251 binds to N-acetylglucosaminyl-1-phosphotransferase α and β (GNPTAB) and anchors it in cis-Golgi, whereas loss of TMEM251 results in GNPTAB mis-trafficking to lysosomes and subsequent degradation. Importantly, TMEM251 knockout mice phenocopies one LSD Mucolipidosis Type II (MLII), suggesting the key role of this gene in maintaining lysosomal homeostasis.

In a parallel study, Pechincha *et al*. used pancreatic adenocarcinoma cell lines to explore the factors that enable cells to use extracellular proteins as nutrients [5]. They first established the defined nutrient conditions where the growth of cancer cells relies on extracellular proteins, and then performed doxycycline-inducible proliferation-based CRISPR screening. Knockout of TMEM251 does not affect cell proliferation under standard culture conditions, but significantly affects cell survival and growth when cancer cells feed on extracellular proteins. Mechanistically, consistent with Richards’s study, they also found that TMEM251 directly interacts with GNPTAB and retains GNPTAB in the Golgi apparatus, which is critical for regulating M6P modification onto the lysosomal proenzymes. Without TMEM251, GNPTAB is degraded by lysosomes due to its hydrophilic transmembrane domain, leading to the hypersecretion of lysosomal enzymes and inhibition of cancer cell proliferation. This study suggests that inhibition of TMEM251 may be a promising strategy to suppress metabolic adaptations in cancer. Due to the important role of TMEM251 in M6P signaling pathway, these two groups renamed this protein as *lys*osomal *e*nzyme *t*rafficking *f*actor (LYSET).

Interestingly, another study from Li’s group also identified TMEM251 as a critical regulator of M6P modification [6]. They performed genome-wide CRISPR/Cas9 screening in HEK293 cells to investigate factors that regulate lysosome function. The authors firstly generated a reporter cell line [GFP-RNF152 (one lysosomal membrane protein)-IRES-mCherry]. Upon cycloheximide treatment, GFP-RNP152 is constitutively degraded via lysosome, while mCherry keeps stable. However, when lysosome function is defective, GFP-RNP152 will be stabilized and the GFP/mCherry ratio is increased. Their screening also identified TMEM251 as an essential factor for lysosomal degradation. Ablation of TMEM251 promotes lysosome biogenesis evidenced by the increased size and number of lysosomes, possibly due to compensatory responses for lysosome dysfunction. More in-depth study showed that TMEM251 knockout promotes the secretion of most lysosomal enzymes without M6P modification into medium, which is consistent with the earlier two studies [4, 5]. Importantly, media containing normal M6P-modified enzymes can rescue the lysosome defects caused by TMEM251 knockout, reinforcing the result that TMEM251 is a key upstream regulator for M6P tagging lysosomal enzymes. Reciprocal IP revealed that TMEM251 co-localizes with GNPTAB. Moreover, the authors found that TMEM251 also interacts with S1P and promotes the cleavage of GNPTAB by S1P which is an essential step for GNPTAB activation. Thus, they renamed TMEM251 as *G*NPTAB *c*leavage and *a*ctivity *f*actor (GCAF). TMEM251 knockout zebrafish display similar clinical features of MLII.

**Figure 1:**
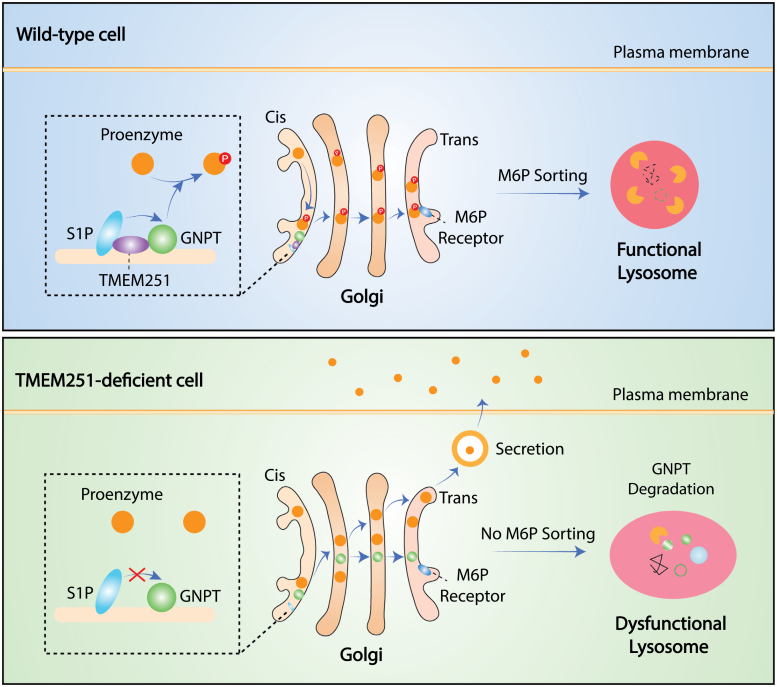
Critical role of TMEM251 in lysosomal enzyme trafficking. At the cis-Golgi, TMEM251 binds to GNPT and S1P, which retains GNPT in the Golgi apparatus and possibly facilitates its cleavage by S1P. Then, GNPT transfers the lysosomal trafficking signal M6P onto the lysosomal proenzymes. Next, these proenzymes are recognized and sorted by MPRs, and trafficked to lysosomes. When TMEM251 is deficient, GNPT is mis-trafficked to lysosomes and subsequent degradation, leading to loss of M6P modification and hypersecretion of lysosomal proenzymes. Dysfunctional lysosomes without active enzymes are abnormally enlarged and filled with undigested materials.

Altogether, these three studies independently identified TMEM251 as an indispensable factor for lysosomal enzyme trafficking (Fig.1). Defective TMEM251 is possibly the underlying mechanism for lysosome-related diseases such as LSDs and cancer. However, the exact molecular mechanism of how TMEM251 regulates GNPTAB activation remains controversial: through stabilization [5] or through cleavage [6]? In addition, the upstream regulators or modulator of TMEM251, if any, remain obscure. Moreover, since lysosome dysfunction is often cross-linked with autophagy, it is worthy to explore the role of TMEM251 in autophagy-related diseases such as neurodegenerative disorders.
